# Worsened self-rated health in the course of the COVID-19 pandemic among older adults in Europe

**DOI:** 10.1093/eurpub/ckad143

**Published:** 2023-08-11

**Authors:** Daniel Lüdecke, Olaf von dem Knesebeck

**Affiliations:** Institute of Medical Sociology, University Medical Center Hamburg-Eppendorf, Hamburg, Germany; Institute of Medical Sociology, University Medical Center Hamburg-Eppendorf, Hamburg, Germany

## Abstract

**Background:**

Governments across Europe deployed non-pharmaceutical interventions to mitigate the spread of coronavirus disease 2019 (COVID-19), which not only showed clear benefits but also had negative consequences on peoples’ health. Health inequalities increased, disproportionally affecting people with higher age or lower education. This study analyzed associations between social factors and worsened self-rated health of elderly people in the course of the COVID-19 pandemic, taking different stringencies of government mandates as well as infection rates into account.

**Methods:**

Data stem from the European SHARE survey. The main outcome was a binary indicator of worsened self-rated health. Analyses included data from two waves (2020 and 2021) during the pandemic (*N* = 48 356 participants, N = 96 712 observations). Predictors were age, sex, education and living together with a partner, and two macro indicators that reflected the stringency of government response mandates and COVID-19 infection rates. Data were analyzed using logistic mixed-effects regression models.

**Results:**

Older age [odds ratio (OR) 1.73, confidence interval (CI) 1.65–1.81] and female sex (OR 1.26, CI 1.20–1.32) were positively associated and higher education (OR 0.74, CI 0.70–0.79) was negatively associated with worsened self-rated health. Not living together with a partner showed higher odds of worsened self-rated health (OR 1.30, CI 1.24–1.36). Inequalities increased from 2020 to 2021. Associations between worsened self-rated health and government response mandates or infection rates were inconsistent.

**Conclusion:**

Self-rated health worsened in the course of the pandemic and health disparities increased. Possible future pandemics require targeted interventions to minimize adverse health outcomes, in particular among old, potentially isolated, and deprived people.

## Introduction

Coronavirus disease 2019 (COVID-19) was an outbreak officially declared as an international public health emergency by the World Health Organization.^1^ In response to the outbreak, governments in Europe and around the world have deployed a wide range of non-pharmaceutical interventions (NPIs) to mitigate the spread of COVID-19. The restrictive mandates imposed varied from a few to assertive approaches of stringent NPIs.^2^ Over the course of the pandemic, there were large differences in terms of infection rates and in the stringency of government responses between countries across Europe and worldwide, with usually lower infection rates in those countries that imposed more stringent NPIs.^3^

NPIs showed clear benefits, were able to reduce the pandemic spread and thereby lowered COVID-19 mortality rates.^4^ Yet, there were also disadvantages. The impact of NPIs on peoples’ social life was often accompanied by negative consequences for health and subjective well-being, which seemed not to be sufficiently considered when NPIs were initially imposed. Different studies also showed that due to COVID-19-related restrictions on physical and social activities, SRH worsened, especially among older adults.^5^^,^^6^

Not only SRH was affected by the outbreak, health inequalities also increased over the course of the pandemic. In this regard, social factors such as education, age, sex, social contacts or living with partner played a significant role. Especially older people with lower educational status were affected by the consequences of NPIs or COVID-19 infections. Studies suggest that worsened SRH was more pronounced for people with lower education or income.^7^^,^^8^ Negative impact of reduced social contacts on SRH could be better compensated by people with higher educational levels.^9^ Considering the elderly population specifically, not only reduced social contacts, but especially living alone without a partner put the elderly at higher risks of negative health outcomes and worsened SRH.^10^ Finally, female sex was associated with worsened SRH, not only physical but also particularly mental health.^11^

Further important factors related to SRH were infection rates and whether one was personally affected by a COVID-19 infection. Widespread occurrences of infectious diseases were closely related to symptoms of psychological distress, such as depression and anxiety, which had negative effects on SRH among older people.^12^^,^^13^ Not only the perceived threat but also the actual COVID-19-affectedness played a role with regard to SRH. Studies showed that short- and long-term negative health consequences can result from COVID-19 infections.^14^^,^^15^

Although there is growing evidence on the links between social factors (such as age, educational level, living with partner or sex) and SRH in the context of the COVID-19 pandemic, little is known about the interplay between these factors and the extent of NPIs. Most studies focus on the associations of social factors and SRH without considering that these associations may differ depending on the intensity of NPIs. Comparisons between countries that deployed different levels of restrictions are often missing. Moreover, the impact of countries’ infection rates has rarely been studied. Hence, while there is increasing research on social factors on an individual level and their associations with SRH, contextual factors on a macro level, such as stringency of NPIs or infection rates, were often not explicitly investigated together with individual-level factors. Finally, as most studies conducted were cross-sectional, there is a lack of studies that analyzed how associations between social factors and SRH change over the course of the pandemic. Therefore, this study aims to address the following research questions: (1) What is the prevalence of worsened SRH among older adults in different European countries during the COVID-19 pandemic and has this prevalence changed in the course of the pandemic? (2) What are the associations of social factors (sex, age, education and living with partner) and COVID-19-affectedness with worsened SRH? (3) Are infection rates and government response mandates associated with worsened SRH? (4) Have these associations changed over the course of the pandemic?

## Methods

### Sample and participants

Analyses were based on data from the eighth and ninth waves of SHARE, the Survey of Health, Ageing and Retirement in Europe.^16^ Only in these two waves special COVID-19 survey data were available. The SHARE survey was conducted in 27 European countries and Israel (see table 1). Probability sampling approaches were used within countries, based on population registers and included non-institutionalized adults aged 50 years or older and, if available, their partners. A multistage stratified sampling design was used in most countries. In countries where respective sampling frames were not available, clustering strategies were applied.^17^ Exclusion criteria were as follows: being incarcerated, moved abroad, unable to speak the language of questionnaire, deceased, hospitalized, moved to an unknown address or not residing at the sampled address.^18^ The data collection for the COVID-19 surveys started in June 2020 for wave 8 and in June 2021 for wave 9, respectively. Data collection ended about 3–4 months later. Hence, these periods took place after an increased occurrence of COVID-19 infections and NPI mandates. As we were interested in worsened SRH in the course of the pandemic, we only considered those respondents who participated in both waves. Thus, for the present study, the final sample size was *N* = 96 712 observations (48 356 unique participants).

**Table 1:** Sample description of respondents, SHARE data (8th wave, 2020), unweighted

Country	*N*	Aged 70+, %	High education, %	Female sex, %	Partner in household, %
**Countries with low Stringency Index (lower tercile)**					
Bulgaria	692	45.5	16.3	60.5	67.3
Croatia	1882	42.2	16.5	56.4	76.2
Czech Republic	2040	60.6	16.7	63.1	63.1
Denmark	1496	44.8	50.7	56.4	73.5
Estonia	4006	54.5	25.9	63.5	58.9
Finland	1279	43.3	41.7	55.3	76.0
Latvia	948	45.3	27.3	63.5	60.1
Lithuania	1229	43.5	39.2	63.5	61.0
Luxembourg	851	40.2	24.2	55.1	77.7
Malta	763	45.1	5.7	56.0	80.2
Slovakia	882	26.3	8.0	55.0	75.1
Switzerland	1727	57.6	19.8	55.0	69.4
Total	17 795	48.5	25.3	59.4	67.8
**Countries with middle Stringency Index (middle tercile)**					
Austria	2278	62.8	28.9	60.8	61.9
Belgium	3357	48.0	39.6	57.0	66.9
Hungary	843	51.0	19.8	61.9	67.9
Netherlands	705	51.9	36.3	55.2	71.9
Poland	2702	38.3	13.8	57.1	74.9
Romania	1448	37.2	5.6	57.5	72.4
Slovenia	2910	51.9	17.1	59.0	72.6
Sweden	946	66.2	41.3	56.6	72.1
Total	15 189	49.7	25.5	58.2	69.8
**Countries with high Stringency Index (upper tercile)**					
Cyprus	609	58.6	15.8	59.4	78.2
France	1807	52.7	29.8	59.8	64.1
Germany	2021	48.1	34.1	54.2	75.1
Greece	3351	50.4	20.5	57.9	72.8
Israel	1254	64.6	41.2	59.4	70.1
Italy	3317	54.3	9.1	56.2	77.5
Portugal	1039	55.6	13.6	56.8	75.8
Spain	1774	64.0	11.5	58.1	70.6
Total	15 172	*54.7*	*21.0*	*57.4*	*73.0*
**Overall**					
SHARE Sample Wave 8^a^	48 156	50.8	24.0	58.4	70.1

a 200 missing cases in Wave 8.

### Additional data sources

We used additional data sources to build two macro indicators. The stringency in government response towards the COVID-19 outbreak was based on the Oxford COVID-19 Government Response Tracker (OxCGRT), a global panel database of pandemic policies.^19^ For each country at each day of the pandemic, mandates to reduce the spread of the outbreak, like lockdowns, reduced social contacts, and similar, were recorded and a daily index of government response for each country (stringency index) was calculated. Countries with higher stringency index have imposed more intensive mandates to reduce the spread of COVID-19. Data on the numbers of confirmed cases of people infected with COVID-19 were taken from the COVID-19 Data Repository by the Center for Systems Science and Engineering at Johns Hopkins University.^20^ Based on these data, the percentages of the population infected with COVID-19 in the course of the pandemic were calculated (described below).

### Measures

#### Dependent variable

In both SHARE waves 8 and 9, worsened SRH was assessed. SRH is a well-accepted measure that reflects objective health conditions and morbidity quite consistently.^21^ In wave 8, this was measured by asking the question ‘If you compare your health with that before the outbreak of Corona, would you say your health has improved, worsened, or stayed about the same?’. A slightly different wording was used in the ninth wave: ‘If you compare your health now to three months ago, would you say your health has improved, stayed about the same, or worsened?’. Although the question texts slightly differed, the temporal references were comparable. Generally, both questions referred to the change in health in the course of the pandemic. Possible answers to both questions were ‘improved’, ‘about the same’ and ‘worsened’. The first two categories were combined, resulting in a binary variable indicating ‘improved or about the same health’ vs. ‘worsened health’.

#### Independent variables

Social factors included in the analyses were respondent’s age, sex, education and living together with a partner. Age was grouped into younger adults (50–69 years old) and older adults (70 years and older). Education was based on the International Standard Classification of Education (ISCED-97),^22^ which ranges from 0 to 6 (low to higher education) and is recoded into three levels: ‘low (lower/upper secondary)’, ‘mid (post-secondary)’ and ‘high (tertiary)’. Furthermore, a variable was generated, based on questions about whether respondents had been affected by a COVID-19 infection or not. This variable had four categories: ‘never been affected by COVID-19’, ‘tested positive, but no symptoms’, ‘COVID-19-illness symptoms’ and ‘hospitalized due to COVID-19’.

The stringency index and the percentage of the population infected with COVID-19 were used as macro indicators in the analyses. For each country, the mean value for the daily Oxford stringency index values, from the start of the pandemic until the beginning of the data collection of SHARE wave 9 (June 2021), was calculated. This variable represented the average government response to the COVID-19 outbreak within each country and had a possible range from 0 to 100.^19^ An indicator was built, with countries being classified into three groups (based on terciles of this variable): low stringency, middle stringency and high stringency. The proportion of the population infected with COVID-19, which was calculated based on the data from the COVID-19 Data Repository, refers to the cumulative percentage at the beginning of the SHARE data collection in each country.

Finally, to see how the prevalence of worsened SRH changed over time, a variable indicating the two time points of data collection during 2020 and 2021 (‘wave’) was included.

#### Statistical analysis

Descriptive statistics were reported separately, first, for social factors and second, for COVID-19-affectedness and worsened SRH. Social factors (age, education, sex, living with partner in the same household) were considered as time-invariant, because their change from waves 8 to 9 was identical for all respondents. As such, these sample characteristics were reported for the initial wave 8 only. Characteristics that varied over time between respondents (COVID-19-affectedness and worsened SRH) were reported for both waves.

To estimate the associations between worsened SRH and our predictors of interest, three mixed-effects logistic regression models for repeated measures were calculated. The first model only included social factors and COVID-19-affectedness, the second model only included the two macro indicators, and the third (full) model included both social factors, COVID-19-affectedness and macro indicators. Time trends were analyzed using interactions between wave and each social factor and COVID-19-affectedness, resulting in five regression models with interaction terms (wave × age, wave × sex, wave × education, wave × partner in same household and wave × COVID-19-affectedness). We calculated predicted probabilities to show the associations between interactions of the predictors and the outcome variable. Pairwise comparisons were used to test differences between groups for statistical significance. For all models, ‘wave’ was used as a random slope, to account for variation over time. Regional variation was taken into account using ‘country’ as a level-2 predictor. Longitudinal post-stratification weights were rescaled for use with mixed models.^23^ Checks for multicollinearity revealed no collinearity issues. Due to the small proportion of missing data, these were not imputed. All analyses were conducted using the R language for statistical computing version 4.2.1.^24^

## Results

### Sample description and prevalence of worsened SRH

The sample description of social factors refers to all respondents at the first time point of data collection (wave 8, table 1). The mean age of respondents was 70.3 years with 50.8% being 70 years or older. 24% had high education. 58.4% were female. 70.1% of the participants were living with their partners in the same household.

Until the end of data collection of wave 8 (June 2020), 97.9% of all respondents across countries were never affected by COVID-19 (table 2). This rate was reduced to 91.6% in wave 9 (June 2021) with a considerable range between countries (80.6% in Bulgaria; 96.6% in Germany and Greece). In terms of research question 1, table 2 shows that more respondents indicated worsened SRH in 2021 (14.5%; range: 5.3% in Denmark to 14.4% in Lithuania), compared with the year before (8.7%; range: 4.8% in Denmark to 25.9% in Israel). This increase in worsened SRH was found in almost all European countries. Only a few Scandinavian countries showed a decline in worsened SRH.

**Table 2:** Sample description of characteristics that changed over time, by wave, SHARE data (8th and 9th wave), unweighted

Country	Wave 8 (2020)						Wave 9 (2021)					
	*n*	COVID affected, %				Worsened health, %	*n*	COVID affected, %				Worsened health, %
		Never	Tested positive (no symptomps)	Symptoms	Hospitalized			Never	Tested positive (no symptomps)	Symptoms	Hospitalized	
**Countries with low Stringency Index (lower tercile)**												
Denmark	1496	97.0	0.6	2.3	0.1	5.3	1494	96.0	0.5	3.1	0.4	4.8
Switzerland	1727	97.1	0.2	2.5	0.2	6.8	1727	93.3	1.3	4.3	1.0	11.0
Czech Republic	2040	98.5	0.0	1.4	0.1	8.1	2042	84.8	1.2	11.6	2.4	14.6
Luxembourg	851	97.2	0.5	2.2	0.1	8.7	850	90.2	1.4	7.8	0.6	12.1
Estonia	4006	98.4	0.1	1.4	0.1	6.2	4003	91.2	0.9	6.8	1.1	15.4
Croatia	1882	99.9	0.0	0.1	0.0	9.9	1884	89.2	1.3	8.6	0.9	17.5
Lithuania	1229	98.6	0.2	1.1	0.1	14.4	1229	88.5	0.4	9.4	1.7	20.9
Bulgaria	692	99.0	0.0	0.7	0.3	7.4	691	80.6	5.2	9.1	5.1	24.7
Finland	1279	97.0	0.0	3.0	0.0	9.1	1281	93.7	0.1	5.9	0.3	8.7
Latvia	948	97.6	0.5	1.9	0.0	7.1	949	92.9	0.6	5.6	0.8	20.1
Malta	763	97.1	0.3	2.5	0.1	8.9	765	93.1	1.3	4.2	1.4	18.2
Slovakia	882	99.3	0.3	0.2	0.1	6.8	882	89.6	0.7	8.7	1.0	14.3
Total	17 795	98.1	0.2	1.6	0.1	7.9	17 797	90.5	1.1	7.2	1.3	14.6
**Countries with middle Stringency Index (middle tercile)**												
Sweden	946	91.9	0.4	7.3	0.4	7.5	944	90.7	0.5	7.9	0.8	8.4
Netherlands	705	96.9	0.0	3.0	0.1	7.1	705	92.5	1.1	6.0	0.4	8.9
Belgium	3357	96.1	0.2	3.3	0.4	9.4	3353	91.2	1.4	5.8	1.6	13.1
Poland	2702	99.3	0.0	0.5	0.2	10.7	2700	83.3	1.9	13.0	1.9	20.3
Hungary	843	99.4	0.1	0.4	0.1	5.6	843	91.5	0.6	6.9	1.1	11.4
Slovenia	2910	98.2	0.0	1.6	0.1	5.8	2910	86.4	0.8	11.3	1.6	13.4
Romania	1448	99.2	0.2	0.2	0.4	7.9	1448	91.6	1.2	5.2	2.0	16.8
Austria	2278	97.9	0.3	1.5	0.2	10.9	2277	95.0	0.4	3.9	0.7	14.3
Total	15 189	97.6	0.2	2.0	0.3	8.6	15 180	89.5	1.1	8.0	1.4	14.4
**Countries with high Stringency Index (upper tercile)**												
Germany	2021	97.5	0.3	2.1	0.0	8.9	2018	96.6	0.4	2.3	0.7	13.4
Spain	1774	97.6	0.1	2.0	0.3	10.5	1769	92.8	1.2	4.7	1.4	17.1
Italy	3317	98.6	0.3	1.0	0.1	9.5	3318	93.9	1.4	3.7	1.0	14.0
France	1807	94.2	0.2	5.3	0.3	11.5	1807	94.5	1.2	3.5	0.8	11.8
Greece	3351	99.3	0.1	0.7	0.0	7.1	3345	96.6	0.2	2.5	0.7	10.1
Israel	1254	97.8	0.0	2.2	0.1	13.8	1255	94.7	0.6	3.9	0.9	25.9
Portugal	1039	98.7	0.2	0.9	0.2	12.7	1037	95.3	0.5	3.6	0.7	18.2
Cyprus	609	98.7	0.7	0.7	0.0	9.2	610	93.4	1.3	4.1	1.1	15.7
Total	15 172	97.9	0.2	1.8	0.1	9.8	15 159	94.9	0.8	3.4	0.9	14.5
**Overall**												
SHARE sample^a^	48 156	97.9	0.2	1.8	0.2	8.7	48 136	91.6	1.0	6.2	1.2	14.5

a 200 missing cases in Wave 8; 220 missing cases in Wave 9.

For almost all countries (except Croatia), the average stringency index increased over time. Until wave 8, the mean stringency index was 43.2. This increased to 58.8 by the end of wave 9 (Supplementary figure S1).

### Individual and macro-level factors associated with worsened SRH since the COVID-19 outbreak

Regression analysis showed that worsened SRH was more likely in wave 9 compared with wave 8 (model 1, table 3). With respect to social factors (research question 2), older age [odds ratio (OR) 1.73] and female sex (OR 1.26) were significantly associated with worsened SRH. People with middle or higher education were less likely to report worsened SRH (OR 0.81 and OR 0.74, respectively), while the odds of worsened SRH increased for respondents who did not live together with their partner in the same household (OR 1.30). COVID-19-affectedness was generally increasing odds that the respondents’ health has worsened, with ORs ranging from 1.62 for participants that tested positive (without symptoms) to 3.77 for those participants who were hospitalized. Regarding macro indicators (research question 3, model 2), there were no statistically significant associations with stringency index or infection rates. Results of model 3 were very similar to those of the first two models, i.e. we found statistically significant associations between social factors and COVID-19-affectedness, while the macro indicators showed weak and inconsistent associations with worsened SRH.

**Table 3:** Logistic mixed models regression, predictors of worsened self-rated health, SHARE waves 8 and 9, OR, 95% CI, and significances (*p*)

	Model 1			Model 2			Model 3		
Predictors	OR	95% CI	*P*	OR	95% CI	*P*	OR	95% CI	*P*
(Intercept)	0.06	0.06–0.07	<0.001	0.08	0.07–0.10	<0.001	0.06	0.05–0.07	<0.001
Wave									
8, 2020 (reference)	1.00	–	–				1.00	–	–
9, 2021	1.56	1.38–1.77	<0.001	1.61	1.40–1.85	<0.001	1.57	1.39–1.77	<0.001
Age, years									
50–69 (reference)	1.00	–	–				1.00	–	–
70+	1.73	1.65–1.81	<0.001				1.73	1.65–1.81	<0.001
Sex									
Male (reference)	1.00	–	–				1.00	–	–
Female	1.26	1.20–1.32	<0.001				1.26	1.20–1.32	<0.001
Education									
Low (reference)	1.00	–	–				1.00	–	–
Middle	0.81	0.77–0.86	<0.001				0.81	0.77–0.86	<0.001
High	0.74	0.70–0.79	<0.001				0.74	0.70–0.79	<0.001
Partner in household									
Yes (reference)	1.00	–	–				1.00	–	–
No	1.30	1.24–1.36	<0.001				1.30	1.24–1.36	<0.001
COVID									
Never been affected (reference)	1.00	–	–				1.00	–	–
Tested positive, no symptoms	1.62	1.31–2.01	<0.001				1.62	1.31–2.01	<0.001
Symptoms	1.92	1.76–2.09	<0.001				1.92	1.76–2.09	<0.001
Hospitalized	3.77	3.13–4.54	<0.001				3.77	3.13–4.55	<0.001
Stringency Index									
Low (reference)				1.00	–	–	1.00	–	–
Middle				1.00	0.76–1.31	0.986	0.93	0.70–1.24	0.631
High				1.12	0.86–1.47	0.396	1.09	0.83–1.45	0.534
% infected									
Lower tercile (reference)				1.00	–	–	1.00	–	–
Middle tercile				1.08	0.90–1.30	0.410	1.06	0.89–1.27	0.480
Upper tercile				1.14	0.94–1.39	0.187	1.11	0.92–1.33	0.269
Observations	88 705			96 292			88 705		

### Changes in associations of social factors and COVID-19-affectedness with worsened SRH between SHARE waves

**Figure 1:**
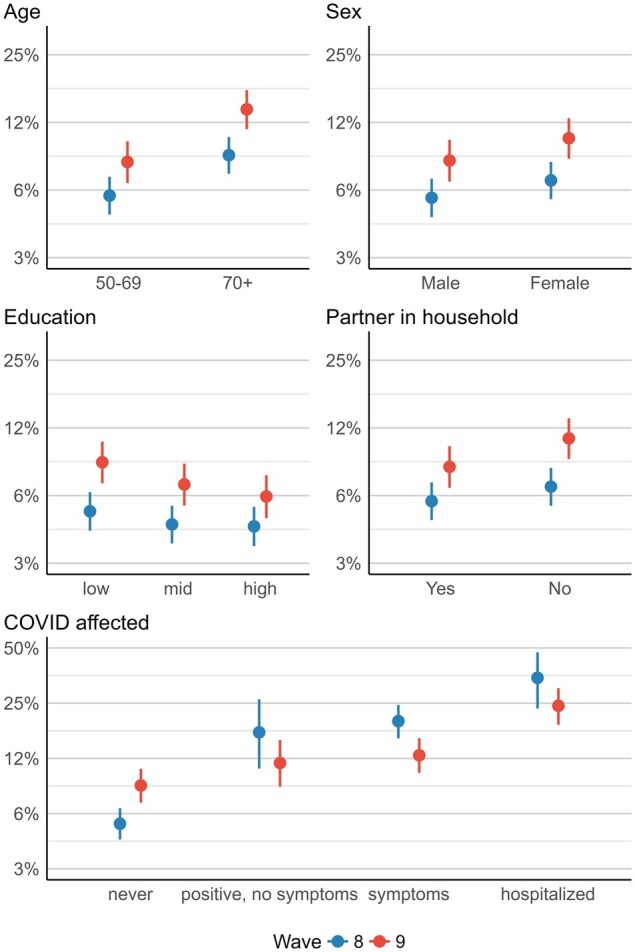
Predicted probabilities of “worsened self-rated health” for interaction terms, showing the change between SHARE wave 8 and 9 (2020–21). Wave 8 = blue, wave 9 = red

When looking at the predicted probabilities for waves 8 and 9 (research question 4, figure 1 and Supplementary table S1), we see that worsened SRH was generally reported more often in wave 9, with stronger increases for people aged 70 years or older, females, low educated respondents and those who did not live together with their partner. For all these social factors, the differences between waves 8 and 9 were statistically significant. In terms of COVID-19-affectedness pattern was different: worsened SRH was more likely in wave 8 when individuals had symptoms, tested positive without symptoms or had to be hospitalized. Only respondents who were never affected by COVID-19 reported worsened SRH in wave 9. Differences between the waves were only statistically significant for individuals who were never affected by COVID-19 and those who had COVID-19 symptoms.

## Discussion

The present study sought to examine the associations of social factors (sex, age, education and living with partner) and COVID-19-affectedness with worsened SRH and whether the macro-level factors of infection rates and NPIs contribute to worsened SRH.

### Summary of main findings

One finding of this study was that worsened SRH among older adults became more prevalent over the course of the pandemic across most European countries, with few exceptions in some Scandinavian countries (research question 1). Another major finding was that all social factors and COVID-19-affectedness were significantly associated with worsened SRH (research question 2). The two macro indicators, stringency index and infections rates, however, showed rather inconsistent and very weak, non-significant associations with worsened SRH (research question 3). Moreover, disparities in SRH increased in the course of the pandemic in particular for people who were not living with a partner in the same household or who had a low educational status (research question 4). Increased worsened SRH could also be found, but less pronounced, for female sex and being 70 years and older.

### Interpretation

The concept of SRH is not strictly limited to physical aspects of health. Rather, it also includes mental health and social dimensions.^25^ The increased prevalence of worsened SRH over the course of the pandemic can be partly explained by individual and macro-level factors that will be discussed in the following. Regarding social factors, it was not surprising that older age was associated with a higher risk of worsened SRH. Female persons were more likely to report worsened SRH. However, this seemed unrelated to a possible COVID-19-affectedness, because the proportion of men and women in our sample who were ever affected by a COVID-19 infection was quite similar. This is backed up by previous studies that found that in several European countries, a similar number of male and female persons were affected, but more severe COVID-19-related health outcomes were observed in aged men.^26–28^ We would therefore conclude that sex- and gender-specific differences in worsened SRH were not particularly related to NPIs, but that rather fear and anxiety and their impact on SRH played a more important role in this regard.^29^

In terms of educational status, we found a gradient: The higher the educational status, the less likely it was that SRH would deteriorate. One explanation is that education is a long-known determinant of health inequalities.^30^ The already existing health inequalities were even exacerbated by the pandemic.^31^ Restrictive government mandates in response to the pandemic outbreak had significantly influenced social contacts and physical activities among older adults and more so among those with lower education, which in turn had negative consequences for SRH.^32^ Similarly, living together with a partner in the same household seemed to serve as a protective buffer against worsening SRH, because social ties have an impact on mental and physical health.^33^ This is particularly true for living together with partners^34^ and partly explains why in our study respondents who were not living with their partner in the same household were more likely to show worsened SRH. Findings from other studies are in line with this interpretation and reported that social support and family functioning were consistently positively associated with health and well-being.^35^

COVID-19-affectedness was negatively associated with SRH, with hospitalization having the strongest impact. Fear of COVID-19 infections in general may also play a role in this regard because even for people who tested positive but showed no symptoms, we found rather strong associations with our outcome. The strong associations between hospitalization and worsened SRH highlight the importance of NPIs to lower infection rates and protect vulnerable population groups.

We found no evidence that NPIs were associated with worsened SRH. This was unexpected because other studies found an impact of pandemic-related NPIs on physical and mental health.^3^^,^^36^^,^^37^ Especially stricter government response mandates were associated with a decline in mental health.^11^ Yet, most of those studies were conducted in a single country, comparing pre- and current pandemic situations, while our study includes many different European countries, all of which differed in terms of the scope and intensity of the NPIs. This might be a reason for the inconsistent associations we found between our macro indicators and worsened SRH. Furthermore, since we were looking at a period of about a year, people may have become more used to NPIs over time, thus associations could be less pronounced.

With regard to the change of worsened SRH over time, we found that disparities increased. In particular, there was a more serious deterioration of SRH for lower-educated people and persons without partners in the same household. Again, we would interpret this result in the context of individual resources that positively influence SRH. These resources seem to degrade more quickly in socially disadvantaged people under pandemic conditions. In the case of lower education, this can additionally be explained by increased prevalence of pre-existing diseases, less favourable health behaviour or other psychosocial risk factors.^38^ However, the associations between COVID-19-affectedness and worsened SRH weakened over the course of the pandemic. This might be related to the fact that fear of COVID-19 diminished, in particular when vaccination became available.

### Strengths and limitations

Some methodological limitations have to be taken into account. The SHARE data only provides some rather crude measures of worsened SRH. The question used at the beginning of the outbreak (wave 8) had a clearer reference to COVID-19 as compared with the worsened SRH in the later SHARE wave. More than 1 year later, a change in SRH may have several causes beyond the impact of NPIs or the COVID-19 context.

Furthermore, the stringency index includes various mandates, such as school closures, gathering restrictions or cancelling public events, which have been implemented differently across countries. During the course of the pandemic, government responses have also changed a lot. Thus, not all components of NPIs affect SRH in the same way. This might explain unsystematic differences in the stringency index and the inconsistent results we found concerning our macro indicators, which revealed no clear pattern regarding associations with SRH.

Moreover, many other factors influence the impact of a pandemic outbreak on SRH. For instance, the SHARE data did not include measures such as job security, loneliness or other psychosocial factors.

Nonetheless, one of the strengths of this study is the combination of a large dataset on an individual level combined with well-prepared data on a macro level from other sources, which allowed us to gain more specific insights into the complex associations of social factors, infection rates, NPIs and worsened SRH. The possibility to include these macro-level data is relatively new and to the best of our knowledge, only a few studies used these data sources so far.^39^ Moreover, by including different time points, we were able to analyze associations with SRH over the course of one year of the pandemic.

## Conclusions

Results indicate that the longer the COVID-19 pandemic lasts, the stronger its negative impact on the SRH of older adults. We could not find clear associations between worsened SRH and different levels of NPIs and restrictions, although this would have been expected. Yet, we found evidence for increasing health inequalities in the course of COVID-19. Thus, for future pandemic events, targeted interventions are needed to minimize adverse health outcomes, in particular among old, potentially isolated, and deprived people.

## Supplementary Material

ckad143_Supplementary_Data

## Data Availability

The SHARE data is available for research purpose after registering at the SHARE website (www.share-project.org). Data from the Oxford COVID-19 Government Response Tracker (OxCGRT) are freely available at https://github.com/OxCGRT/covid-policy-tracker. The COVID-19 Data Repository stores data on COVID cases and are located at https://github.com/CSSEGISandData/COVID-19. All websites were lastly accessed at the 30 April 2023. All source code (in R) to reproduce the data preparation and analysis is available at https://osf.io/cht59/ (DOI 10.17605/OSF.IO/CHT59). Key points There is a lack of studies that analyzed how associations between social factors and self-rated health changed over the course of the COVID-19 outbreak, in particular considering the impact of non-pharmaceutical interventions. Worsened self-rated health among older adults became more prevalent over the course of the pandemic across most European countries and social factors such as age, education, sex and living alone as well as COVID-19-affectedness were significantly associated with worsened self-rated health. Targeted interventions are needed to minimize adverse health outcomes, in particular among old, potentially isolated and deprived people.
