# Supplementary material for: Worsened self-rated health in the course of the COVID-19 pandemic among older adults in Europe
Source: Eur J Public Health. 2023 Aug 11;33(6):1148–54. doi: 10.1093/eurpub/ckad143 (PMC10710346; doi:10.1093/eurpub/ckad143)
Supplement: ckad143_Supplementary_Data [file ckad143_supplementary_data.zip › ckad143_Supplementary_Data/ejph-2022-12-om-0583-File003.pdf]

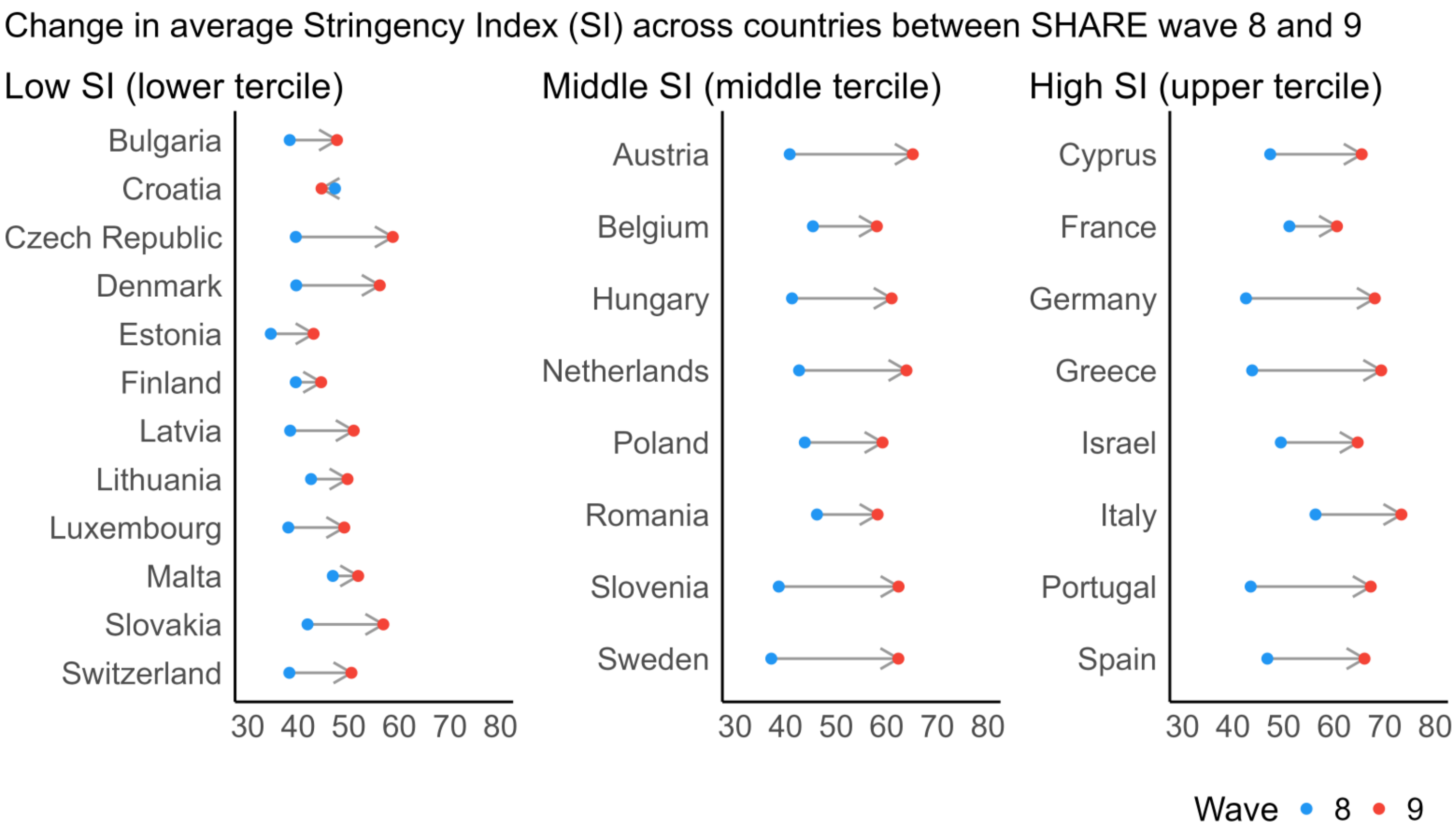

Change in average Stringency Index (SI) across countries between SHARE wave 8 and 9
Low SI (lower tercile)
Bulgaria
Croatia
Czech Republic
Denmark
Estonia
Finland
Latvia
Lithuania
Luxembourg
Malta
Slovakia
Switzerland
30 40 50 60 70 80
Middle SI (middle tercile)
Austria
Belgium
Hungary
Netherlands
Poland
Romania
Slovenia
Sweden
30 40 50 60 70 80
High SI (upper tercile)
Cyprus
France
Germany
Greece
Israel
Italy
Portugal
Spain
30 40 50 60 70 80
Wave 8 9
