# Supplementary material for: Worsened self-rated health in the course of the COVID-19 pandemic among older adults in Europe
Source: Eur J Public Health. 2023 Aug 11;33(6):1148–54. doi: 10.1093/eurpub/ckad143 (PMC10710346; doi:10.1093/eurpub/ckad143)
Supplement: ckad143_Supplementary_Data [file ckad143_supplementary_data.zip › ckad143_Supplementary_Data/ejph-2022-12-om-0583-File004.pdf]

Changes in associations of social factors and COVID-19-affectedness with worsened SRH between SHARE waves

The following table S1 shows the predicted probabilities of worsened SRH and their confidence intervals for the interaction terms. These numbers are the same numbers used to generate Figure 1 in the main text. Additionally, the relative inequalities between the lowest and highest levels of each social factor and COVID-19-affectedness are presented, which indicates whether inequalities have increased (factor > 1) or decreased (factor < 1) between SHARE waves 8 and 9.

The relative inequalities regarding to worsened SRH increased from wave 8 to 9 for age, sex, education and partner in household and decreased for COVID-19 affectedness.

Table S1: Predicted probabilities of "worsened self-rated health" for interaction terms and 95% confidence intervals (CI), comparisons between SHARE wave 8 and 9 (2020 to 2021), and relative inequalities between predictor levels

	Wave 8			***Wave 9***		
Predictors	***Predicted probability (%)***	***95% CI***	***Relative inequality***	***Predicted probability (%)***	***95% CI***	***Relative inequality***
**Age**						
50-69	5.9	4.9 – 7.2	1.5	8.3	6.7 – 10.3	1.7
70+	8.9	7.4 – 10.8		14.3	11.7 – 17.4	
**Sex**						
male	5.8	4.7 – 7.0	1.2	8.5	6.8 – 10.5	1.3
female	6.9	5.7 – 8.3		10.6	8.6 – 13.0	
**Education**						
high	4.6	3.7 – 5.6	1.17	6.2	5.0 – 7.7	1.42
low	5.3	4.4 – 6.5		8.8	7.1 – 10.9	
**Partner in household**						
yes	5.9	4.9 – 7.2	1.16	8.4	6.8 – 10.4	1.34
no	6.9	5.6 – 8.3		11.2	9.1 – 13.8	
**COVID-19-affectedness**						
never been affected	5.5	4.5 – 6.7	6.25	8.9	7.2 – 11.0	2.72
hospitalized	34.4	23.4 – 47.4		24.2	19.1 – 30.2	
